# Identifying and Assessing Foundational Competencies in Geropsychology Supervision

**DOI:** 10.1093/geroni/igaf122.779

**Published:** 2025-12-31

**Authors:** Holly Malette, Jacqueline Gurevitch, Michelle Mlinac

**Affiliations:** VA Boston Healthcare System, Boston, MA, Massachusetts, United States; VA Boston Healthcare System, Boston, Massachusetts, United States; VA Boston Healthcare System, Boston, Massachusetts, United States

## Abstract

While previous generations of older adults were less likely to engage in mental health care, efforts to reduce mental health stigma and the aging of progressive generations will likely increase demand for these services. The geriatric mental health workforce must be equipped to meet this need. The Pikes Peak Geropsychology Knowledge and Skill Assessment Tool outlines and measures competencies for professional practice in geropsychology. These competencies do not include providing geropsychology supervision, yet supervised clinical experiences are integral for developing the knowledge, skills, and attitudes required for independent practice. The aim of this project is to define competencies for the provision of geropsychology supervision and develop a tool for their assessment. Proposed supervision knowledge and skills were generated from both literature review and professional experience. This first version of the competency tool was piloted with geropsychology trainees as part of a didactic seminar series. This second version was disseminated to psychologists involved in training geropsychology students. This group of geropsychologists provided feedback and a qualitative analysis was conducted. Respondents underscored the importance of supervisors competent in geropsychology and also highlighted supervision competencies unique to geropsychological practice (e.g. interprofessional collaboration, managing countertransference and supporting caregivers). While further assessment of the utility and acceptability of this tool is warranted, the eventual goal is to utilize it alongside the Pikes Peak tool to evaluate trainee competencies throughout their training and to guide the supervision practices of geropsychologists.

